# Influence of Growth Process on Suppression of Surface Morphological Defects in 4H-SiC Homoepitaxial Layers

**DOI:** 10.3390/mi15060665

**Published:** 2024-05-21

**Authors:** Yicheng Pei, Weilong Yuan, Yunkai Li, Ning Guo, Xiuhai Zhang, Xingfang Liu

**Affiliations:** 1 Key Laboratory of Semiconductor Materials Science, Institute of Semiconductors, Chinese Academy of Sciences, Beijing 100083, China; peiyicheng@semi.ac.cn (Y.P.); yuanweilong@semi.ac.cn (W.Y.); liyunkai@semi.ac.cn (Y.L.); guoning@semi.ac.cn (N.G.); 2 School of Resources, Environment and Materials, Guangxi University, Nanning 530004, China; 3 College of Materials Science and Opto-Electronic Technology, University of Chinese Academy of Sciences, Beijing 100049, China; 4 Beijing Key Laboratory of Low Dimensional Semiconductor Materials and Devices, Beijing 100083, China

**Keywords:** 4H-SiC, epitaxial, growth process, defect

## Abstract

To address surface morphological defects that have a destructive effect on the epitaxial wafer from the aspect of 4H-SiC epitaxial growth, this study thoroughly examined many key factors that affect the density of defects in 4H-SiC epitaxial wafer, including the ratio of carbon to silicon, growth time, application of a buffer layer, hydrogen etching and other process parameters. Through systematic experimental verification and data analysis, it was verified that when the carbon–silicon ratio was accurately controlled at 0.72, the density of defects in the epitaxial wafer was the lowest, and its surface flatness showed the best state. In addition, it was found that the growth of the buffer layer under specific conditions could effectively reduce defects, especially surface morphology defects. This provides a new idea and method for improving the surface quality of epitaxial wafers. At the same time, we also studied the influence of hydrogen etching on the quality of epitaxial wafers. The experimental results show that proper hydrogen etching can optimize surface quality, but excessive etching may lead to the exposure of substrate defects. Therefore, it is necessary to carefully control the conditions of hydrogen etching in practical applications to avoid adverse effects. These findings have important guiding significance for optimizing the quality of epitaxial wafers.

## 1. Introduction

With the gradual advancement of silicon carbide (SiC) chemical vapor deposition (CVD) technology, the SiC growth process has approached maturity, and the primary obstacle to producing SiC semiconductor epitaxial wafers lie in the presence of epitaxial defects. These defects pose significant challenges for high-voltage and high-power SiC power electronic devices [1,2,3,4,5,6,7]. The origins of these defects are often related to many factors, such as substrate quality, growth temperature and cavity structure, and the crystal structure of these defects is usually complicated [8,9,10,11,12,13,14,15]. High-voltage and high-power SiC power electronic devices need a large active area to realize high-current applications [16,17,18,19,20]. Defects in the active region degrade the performance of devices and further lead to device failure. Especially, some fatal defects can greatly reduce a device’s breakdown voltage and may cause long-term reliability problems [21,22,23]. 

The epitaxial defects of silicon carbide are mainly divided into surface morphology defects and internal structure defects, and internal structure defects are mainly dislocation and some hole-like point defects. Micropipes in structural defects cause serious damage, but they have already been solved [24]. Other structural defects, such as through edge dislocation (TED) and through screw dislocation (TSD), cause surface pits and increase leakage current, which are less destructive. The main destructive defects are instant surface dislocation (BPD) defects and stacking faults (SF), which are likely to continuously increase the on-resistance of bipolar devices [25,26,27,28,29,30,31,32,33,34,35]. Surface defects, such as dump, scratch, particle, downfall (DF), triangle (TD), comet and carrot defects, are typically detrimental and easily observable, and often lead to device failure [36,37,38,39,40,41,42].

Therefore, one of the main challenges in 4H-SiC epitaxial growth is to decrease the defect density of the epitaxial layer. There is less research regarding suppressing SiC epitaxial defect formation using epitaxial process modulation. Therefore, this study is significant. In this study, a multitude of experiments were conducted on different process parameters to examine the impact of different growth conditions on the density of surface defects. Through these effects, defect control methods for growth process control were obtained.

## 2. Experimental

**Figure 1:**
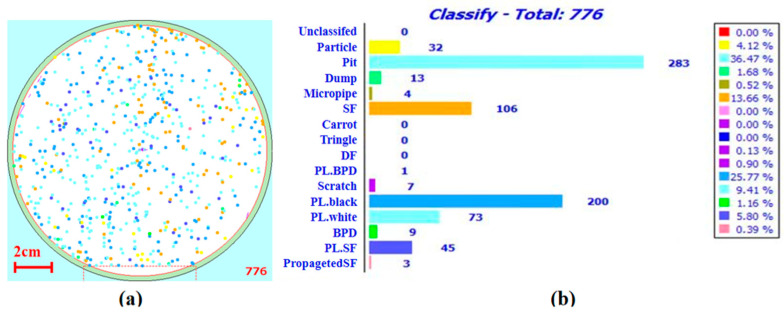
Sample of epitaxial wafer defect test: (**a**) defect distribution and (**b**) defect quantity statistics.

To determine the influence of some process parameters on the density of defects, we used a 4° inclination angle to grow a 6-inch 4H silicon carbide epitaxial wafer. Trichlorosilane (TCS, SiHCl_3_) was used as the silicon source. TCS ensured that the ratio of silicon to chlorine was 1/3, ethylene was used as the carbon source, hydrogen was used as the carrier gas, and the ratio of silicon to hydrogen was 0.05%, which is around 50 sccm TCS and 100 slm H_2_. The conventional conditions were a C/Si ratio of 0.72, temperature of about 1570 °C, pressure of 40 torr, and epitaxial growth for 30 min. The experimental conditions of the target test items were adjusted, while ensuring that other experimental conditions remained unchanged. SICA examination equipment for distinguishing defects using photoluminescence defect morphology was used to detect and count the density of defects to investigate how the density of defects changes under different conditions. The main defects found using this examination are presented in Figure 1. Among them, defects with PL preceding the defect name are those that have not been found under ordinary optical inspection, but only found under photoluminescence examination. PL.white and PL.black are defects such as TED and TSD, and this examination method could not accurately distinguish between these two defects.

## 3. Results

**Figure 2:**
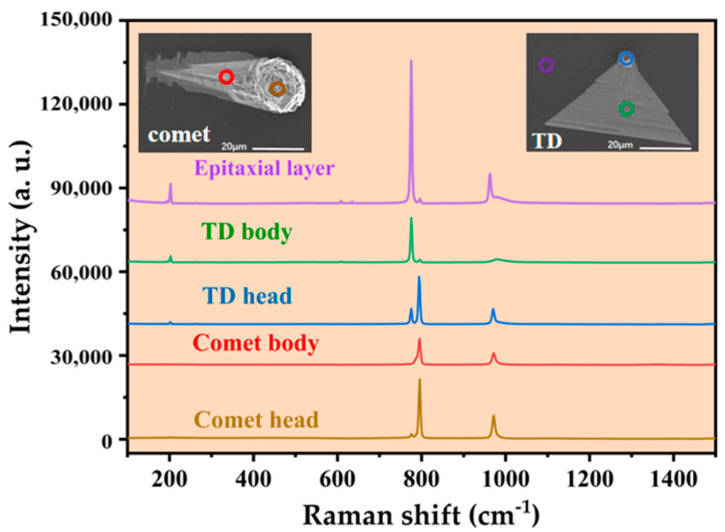
Identification of comet defects and triangle defects by Raman spectroscopy.

Through comparative experiments, the effects of process parameters, such as the C/Si ratio, temperature, growth time, buffer layer and etching conditions, on the density of epitaxial defects of 4H-SiC were identified. A scanning electron microscope (SEM) provides high-resolution images of the surface topography and can be used to analyze the morphology, composition, and crystal structure of materials. The surface of the grown epitaxial wafer was observed by SEM, and the main defects observed are shown in Figure 2. The two defect types are comet defects and triangle defects; comet defects are caused by downfall defects, while triangle defects may be caused by structural defects inherited from the substrate in addition to dropouts [1,27].

Figure 2 shows the Raman examination of two common surface defects. The one with a peak at 777 cm^−1^ is a 4H crystal form, and the one with a high peak at 798 cm^−1^ is a 3C crystal form. Therefore, the head and body of comet defects were both the second phase of the 3C structure, the head of triangular defects was a mixed crystal form dominated by a 3C structure, and the body parts were all 4H crystal forms, like epitaxial layers.

(1) Defect suppression by the C/Si ratio

First, the mainstream process parameter is the ratio of carbon to silicon, which can reflect the growth source. In the experiment, we mainly explored the relationship between the ratio of carbon to silicon and the density of defects in epitaxial wafers, in which the ratio of carbon to silicon was changed by changing the ratio of TCS to ethylene, keeping the amount of TCS unchanged, and reducing the amount of ethylene to reduce the ratio of carbon to silicon. In addition, to explore the influence of the C/Si ratio on the density of defects, the TCS flux was set at 50 sccm, and the ethylene flux was adjusted at 25, 20, 18 and 13, so that the C/Si ratios were 1, 0.8, 0.72 and 0.52, respectively. Under these conditions, the epitaxy was carried out for 30 min.

Only cases in which C/Si ratios were less than 1 were selected because TCS contains chlorine, which generates SiCl4 with a part of Si. To balance the actual C/Si ratio participating in the reaction, a small amount more of the Si source was more suitable for epitaxial growth. Only conditions when the C/Si ratio was 1, 0.8, 0.72 and 0.52 were selected for epitaxial experiments.

**Figure 3:**
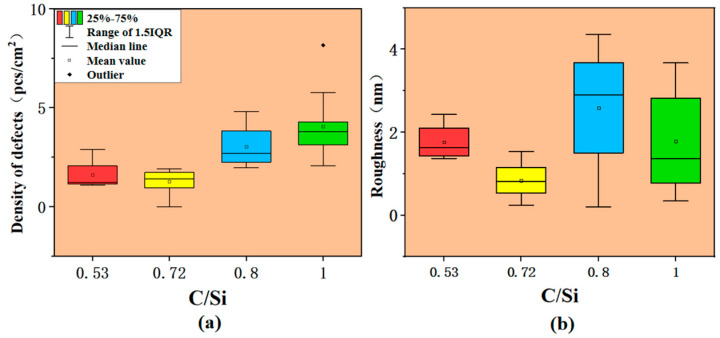
Effect of C/Si ratio on defect and roughness. Red, yellow, blue and green blocks represent sample data range when the C/Si ratio is 0.52, 0.72, 0.8 and 1, respectively: (**a**) density of defects and (**b**) roughness.

In Figure 3a, boxes show the overall situations of defect density under different C/Si ratios, which could obtain the density of epitaxial defects under different C/Si ratios, reflect the discrete nature of defect density data to a certain extent, and obtain the stability of the defect density under these C/Si ratios, i.e., it was not easy to obtain epitaxial wafers with particularly high defects. The effect of C/Si on roughness is shown in Figure 3b, and the results for roughness were close to those for defect density. The optimum value was obtained around C/Si = 0.72, and it can be concluded that the defect density can be better suppressed at this C/Si ratio. According to the analysis of obtained data, the defect density did not increase gradually with the increase in the C/Si ratio, but was obtained when the C/Si ratio was maintained at a low level, and after the C/Si ratio reached 0.8, the defect density greatly improved. It is inferred from the experimental results that an increase in the C/Si ratio led to an increase in defect density to some extent. When the C/Si ratio was 0.72, the sample with the smallest defect density was obtained. The overall defect density was low [42]. On the contrary, samples with a higher defect density than those in the same group appeared in the other three groups of C/Si ratio tests, which may have been caused by the temperature change during the heating process.

**Figure 4:**
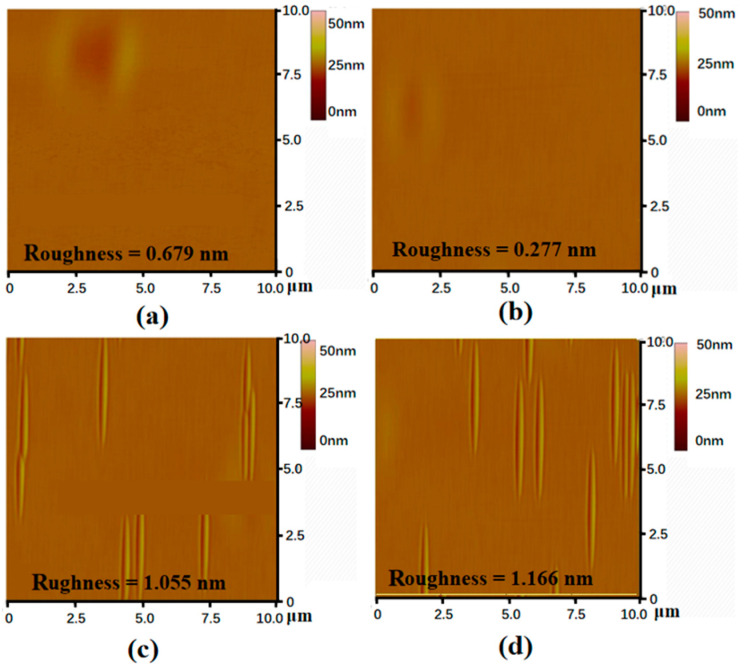
AFM images of epitaxial wafer roughness sampling at different C/Si ratios: (**a**) C/Si = 0.53; (**b**) C/Si = 0.72; (**c**) C/Si = 0.8 and (**d**) C/Si = 1.

Figure 4 shows atomic force microscope (AFM) images taken at different C/Si ratios; an AFM was used to test a plane of 10 μm × 10 μm in tapping mode. Roughness, as shown in Figure 4, was generally used to express the surface quality of epitaxial wafers. However, in the same batch of epitaxy, the main reasons for roughness were pits and steps, as shown in the following atomic force images in Figure 4, which seriously increased the surface roughness. The pits were formed by BPD defects extending to the epitaxial surface. When the C/Si ratio was 0.72, both the roughness and the density of defects were relatively low, which showed that a C/Si ratio of 0.72 is an excellent condition to keep the surface smooth, with fewer defects in process growth.

The reason for this phenomenon may be that the C/Si ratio was affected by the Cl element in the growth source TCS, which hindered the Si elements combining to form silicon drops during SiC epitaxy. On the other hand, under the condition of rich silicon, element C is also difficult to polymerize into carbon particles, and silicon droplets and carbon falling particles are the main generation sites of surface defects, which means that the higher the C content, the easier it is to produce defects. We can control the generation of epitaxial defects to some extent by controlling the ratio of carbon to silicon. After subsequent reference to defect types, defect density, defect distribution, surface roughness, roughness distribution uniformity and various tests, we think that when TCS is used as a Si source for silicon carbide epitaxial wafers growth, there are fewer defects when the C/Si ratio is about 0.72.

(2) Effect of growth time on the density of defects

We studied the influence of growth time on the density of epitaxial defects of silicon carbide, and we thought that growth time was also an important factor affecting the density of defects. At the same time, under conditions of 1570 °C and 40 torr, we controlled the C/Si ratio to be 1 to perform multiple groups of epitaxial growth experiments for 10 min, 20 min, 25 min, 30 min and 40 min, calculated the average density of defects in each group, and plotted the relationship between the density of defects and growth time.

**Figure 5:**
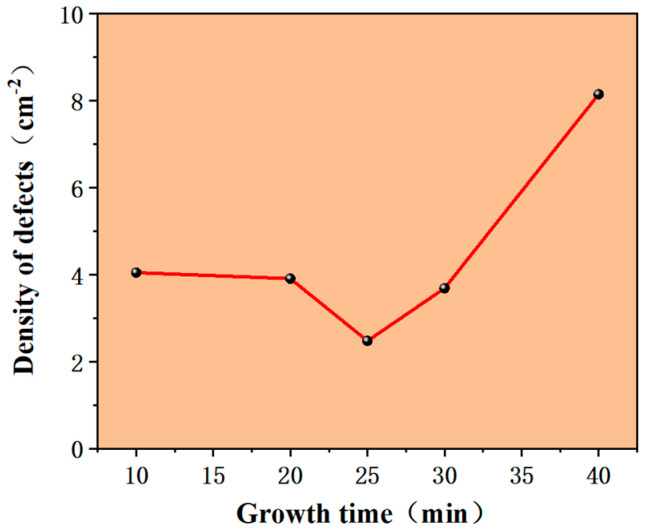
Relationship between the density of defects and growth time.

It can be observed from Figure 5 that the density of defects decreased when epitaxial growth time increased from 10 min to 25 min, and reached a minimum at 25 min, but when epitaxial growth time was adjusted to 40 min, the density of defects increased abnormally compared with the previous states.

The reason for this phenomenon may be that the types of defects were related to the differences in nucleation methods. In the initial stage of growth, defects were mainly structural defects inherited from the substrate or surface defects formed with substrate defects as nucleation sites. During the growth process of 10–25 min, some structural defects inherited from the substrate gradually decreased due to their own transformation and annihilation mechanisms, until they reached a low level at 25 min, during which the transformation from BPD to TED occurred. When the epitaxial time continued to increase to 30 min, the surface morphology defects of downfalls and nucleation of falling particles may have become the mainstream of defects. With the continuous accumulation of defects, the density of defects increased sharply. Therefore, controlling the growth time can be an effective method to reduce the density of defects. This method requires numerous attempts during actual production, focusing on identifying the lowest density of defects growing with time, and then devising production plans accordingly.

(3) Restrain the inheritance of substrate defects via buffer layer

We conducted a control experiment on whether to grow the buffer layer. The growth of the buffer layer occurred under conditions of a C/Si ratio of 0.72, temperature of 1570 °C and pressure of 40 torr. The buffer and epitaxial layers were homogeneous, both 4H-SiC, but the doping concentration of buffer layer was higher than that of the epitaxial layer and lower than that of the substrate. One experiment directly grew the buffer layer for 25 min, and the other experiment grew the buffer layer for 5 min before the same 25 min epitaxy, with a H_2_ flow rate of 100 slm, nitrogen flow rate of 50 sccm, C/Si ratio of 0.53, growth pressure of 40 torr and growth temperature of 1570 °C.

**Figure 6:**
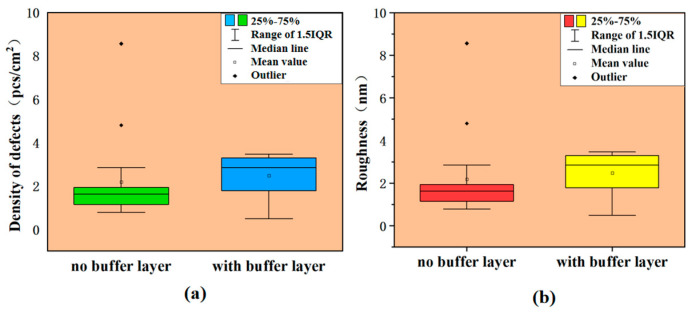
Effect of buffer layer on defects and roughness. Red and green blocks represent the sample data range without buffer layer, while blue and yellow blocks represent the sample data range with buffer layer: (**a**) density of defects and (**b**) roughness.

According to the literature [43], the buffer layer can impede the growth of structural defects into the epitaxial layer with the stress difference brought about by the difference in doping concentration, which can reduce structural defects in the epitaxial layer and surface morphology defects caused by structural defects. Statistical results from experimental data in Figure 6 show that the defect density of epitaxial wafers with a buffer layer was slightly higher than that of epitaxial wafers without a buffer layer, which is contrary to the literature reports [43]. This was because the homogeneous buffer layer was less effective in hindering defects. Instead, the need to grow the buffer layer prolonged the overall epitaxial growth time, making the number of downfall defects higher. 

However, the epitaxial layer shown in Figure 6b with a growing buffer layer had a lower roughness, which may mean that the buffer layer reduced defects that have a large impact on the surface roughness, such as triangle defects.

To verify the effectiveness of the buffer layer on epitaxial defects, we studied serious defects, such as triangular defects, separately. The buffer layer was grown under specific conditions, with a H_2_ flow rate of 100 slm, TCS flow rate of 50 sccm, ethylene flow rate of 25 sccm, growth pressure of 40 torr, growth temperature of 1570 °C and growth time of 4 min. During the experiment, two types of buffer layers were grown; one was a buffer layer with a nitrogen flow rate of 50 sccm, and the other was a buffer layer with a nitrogen flow rate of 300 sccm.

**Figure 7:**
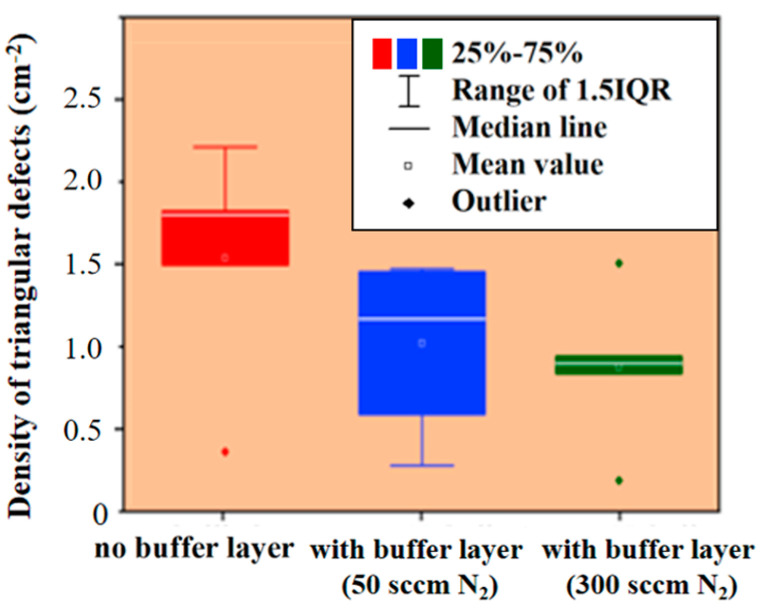
Correspondence between buffer layer and triangle defects. The red, blue and green color blocks respectively represent the sample data range of no buffer layer, with buffer layer of 50 sccm N_2_ flow and with buffer layer of 300 sccm N_2_ flow.

Obviously, the buffer layer grown on the epitaxial wafer plays an important role in triangular defects. We observed more triangular defects on epitaxial wafers without buffer layer growth, and the density of defects decreased significantly after buffer layer growth. This was because the buffer layer prevented some substrate defects from extending to the epitaxial layer and transforming into nucleation sites of surface morphology defects. In addition, the stress and interface defects caused by the doping concentration difference between the substrate and the epitaxial layer can become the nucleation sites of triangular defects, and the nucleation of triangular defects can be inhibited by adding the buffer layer. Therefore, the difference in the effect of buffer layers grown at different nitrogen flow rates, as shown in Figure 7, was due to the difference in doping concentration. Therefore, it can be determined that the buffer layer is extremely effective in controlling the density of defects, and this effect can be achieved by blocking the extension of defects and reducing the concentration difference stress.

(4) Influence of hydrogen etching

Hydrogen etching is the initial stage of the epitaxial process. Its purpose is to expose the high-density atomic steps with narrow mesa width on the (0001) surface of the 4H-SiC epitaxial wafer cut off-axis at 4°, so that 4H-SiC can grow in step flow, which can effectively maintain the crystal form of the epitaxial layer as a 4H structure and improve the uniformity of the epitaxial wafer in all aspects.

Hydrogen etching can enhance the surface thickness uniformity of the epitaxial wafer and reduce the density of defects to some extent. In the experiment, before the epitaxial layers were formally grown, substrates were etched with hydrogen, and etching times were as follows: 0 min, 2 min, 6 min and 12 min.

**Figure 8:**
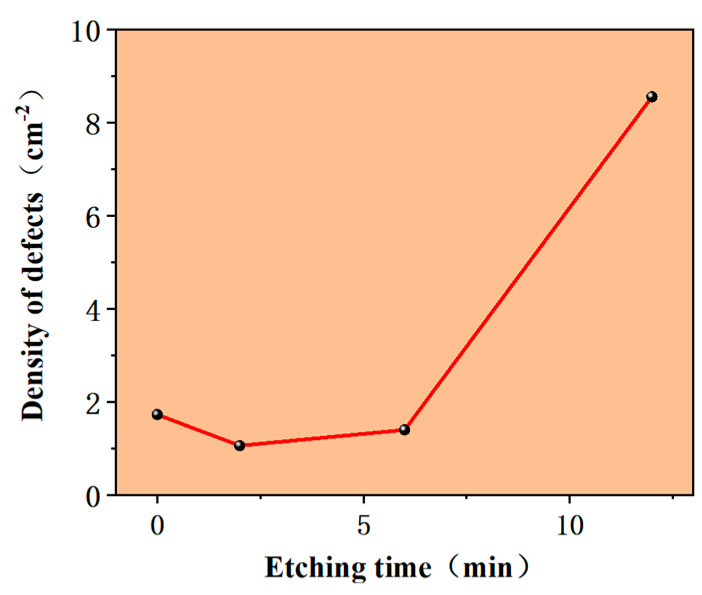
Effect of hydrogen etching time on defect density.

It can be seen in Figure 8 that under the condition of high hydrogen flow etching, the density of defects first decreased and then rapidly increased. The reason for this may be that under the condition of short-time etching, the effect of exposing steps to optimize the growth of surface step flow was achieved, leading to a reduction in the density of defects. However, the hydrogen flow was too large. With the increase in hydrogen etching time, the original defect position of the substrate was not easy to etch because of greater stress, which exposed all substrate defects, and the density of defects inherited by the epitaxial layer from the substrate increased rapidly, resulting in an abnormal increase in the final density of defects. Therefore, hydrogen etching was used to improve the surface energy of the substrate and reduce the density of surface morphology defects. However, care was taken to prevent excessive etching that could expose substrate defects.

## 4. Discussion

This study mainly focused on how to solve the problem of 4H-SiC defects using the epitaxial process. We identified the effects of process parameters, for epitaxial growth, including the C/Si, growth temperature, growth time, buffer layer, and etching conditions, on the density of 4H-SiC epitaxial defects through many experiments.

Various factors affecting the epitaxial defects of 4H-SiC were considered. Combined with the actual defect distribution, we think that the key factors affecting defect density are the C/Si ratio, growth temperature, growth duration, buffer layer and etching strip. The C/Si ratio and growth temperature mainly affected the proportion of elements to control defects. Silicon droplets can become surface defects under silicon-rich conditions, and the proportion of elements affects deep-level defects such as holes. During the growth time, structural defects were the main defects in the early stage, and surface morphology defects were the main defects in the later stage. Controlling the growth time balanced the density of the two defects at a low level. The buffer layer suppressed structural defects and lattice differences caused by excessive variation in doping concentration. Etching can optimize surface quality, but excessive etching exposed the defects of the substrate and greatly increased the density of defects.

However, in fact, the influence of these process parameters on SiC epitaxial defects was interactive and synergistic. For example, shortening epitaxial time inhibited surface morphology defects, but increased structural defects. The proportion of elements affected the extension of structural defects and the concentration of vacancies, and produced silicon droplets or carbon falling objects, resulting in surface morphology defects. It is quite difficult to discuss these composite effects, so we only studied the total defect density. 

## 5. Conclusions

In summary, we conducted systematic research on the influence of process control on SiC epitaxial defects. For defect density, the C/Si ratio was the most influential factor. With an increase in the C/Si ratio from 0.52 to 1, the defect density first decreased and then increased, reaching a lowest level of around 0.72. It reached the lowest defect density when the growth time was about 25 min. The buffer layer effectively reduced the density of surface defects such as triangular defects, but it may have had the opposite effect on other defects. In addition, 100 slm high-flow H_2_ etching for 2–6 min effectively reduced the defect density. Based on the above factors, the defect density of the epitaxial layer decreased by around 60%. Thus, high-flow H_2_ etching played a good role in defect control, especially regarding the density of the serious defects, which was significantly reduced. This defect control method is expected to be beneficial for studies regarding solving SiC defects.

## Data Availability

Data that support the findings of this study are available from the corresponding authors, X.Z. and X.L., upon reasonable request.

## References

[1] Mao W.W., Cui C., Xiong H.F., Zhang N.F., Liu S., Dou M.F., Song L.H., Yang D.R., Pi X.D. (2023). Surface defects in 4H-SiC: Properties, characterizations and passivation schemes. Semicond. Sci. Technol..

[2] Zeng Q., Yang Z., Wang X., Li S., Gao F. (2024). Research Progress on Radiation Damage Mechanism of SiC MOSFETs Under Various Irradiation Conditions. IEEE Trans. Electron Devices.

[3] Xu M., Girish Y.R., Rakesh K.P., Wu P., Manukumar H.M., Byrappa S.M., Udayabhanu, Byrappa K. (2021). Recent advances and challenges in silicon carbide (SiC) ceramic nanoarchitectures and their applications. Mater. Today Commun..

[4] Zhao S.Q., Chen J.H., Yang S.Y., Yan G.G., Shen Z.W., Zhao W.S., Wang L., Liu X.F., Sun G.S., Zeng Y.P. (2023). Effect of temperature on growth of epitaxial layer on semi-insulating 4H-SiC substrate. J. Cryst. Growth.

[5] Zhao S.Q., Wang J.L., Yan G.G., Shen Z.W., Zhao W.S., Wang L., Liu X.F. (2022). Surface Uniformity of Wafer-Scale 4H-SiC Epitaxial Layers Grown under Various Epitaxial Conditions. Coatings.

[6] Zhao Z.F., Li Y., Wang Y., Zhou P., Li Z.H., Han P. (2023). 4H-SiC trench filling by chemical vapor deposition using trichlorosilane as Si-species precursor. J. Cryst. Growth.

[7] Osipov A.V., Grashchenko A.S., Gorlyak A.N., Lebedev A.O., Luchinin V.V., Markov A.V., Panov M.F., Kukushkin S.A. (2020). Investigation of the Hardness and Young’s Modulus in Thin Near-Surface Layers of Silicon Carbide from the Si- and C-Faces by Nanoindentation. Tech. Phys. Lett..

[8] Yang J.W., Song H.P., Jian J.K., Wang W.J., Chen X.L. (2021). Characterization of morphological defects related to micropipes in 4H-SiC thick homoepitaxial layers. J. Cryst. Growth.

[9] Yang G., Luo H., Li J.J., Shao Q.Q., Wang Y.Z., Zhu R.Z., Zhang X., Song L.H., Zhang Y.Q., Xu L.B. (2022). Discrimination of dislocations in 4H-SiC by inclination angles of molten-alkali etched pits. J. Semicond..

[10] Lu P., Huang W., Wang J., Yang H., Guo S., Li B., Wang T., Zhang C., Tu R., Zhang S. (2024). High-throughput thermodynamic study of SiC high-temperature chemical vapor deposition from TMS-H2. J. Cryst. Growth.

[11] Ding W., Lu P., Xu Q., Zhang Z. (2024). Grain size and grain boundary characteristics on the out-plane thermal conductivity of< 111>-oriented CVD 3C-SiC. Ceram. Int..

[12] Mahadik N.A., Stahlbush R.E., Dudley M., Raghothamachar B., Hinojosa M., Lelis A., Sung W. (2023). Formation and propagation mechanism of complex stacking fault in 180 μm thick 4H-SiC epitaxial layers. Scr. Mater..

[13] Xue L.H., Feng G., Liu S. (2022). Molecular dynamics study of temperature effect on deformation behavior of m-plane 4H-SiC film by nanoindentation. Vacuum.

[14] Xue L., Feng G., Wu G., Gao B., Li R., Liu S. (2023). Effect of texture on 4H-SiC substrate surface on film growth: A molecular dynamics study. Vacuum.

[15] Mochizuki K., Mishima T. (2021). Analysis of surface diffusion of carbon- and nitrogen-containing molecules during homoepitaxial growth of 4H-SiC (0001) under silicon-rich conditions. Jpn. J. Appl. Phys..

[16] Huang J.R., Chen T.W., Lee J.W., Huang C.F., Hong L.S. (2022). A perspective on leakage current induced by threading dislocations in 4H-SiC Schottky barrier diodes. Mater. Lett..

[17] Gao W.D., Yang G., Qian Y.X., Han X.F., Cui C., Pi X.D., Yang D.R., Wang R. (2023). Dislocation-related leakage-current paths of 4H silicon carbide. Front. Mater..

[18] Yang S.Y., Zhao S.Q., Chen J.H., Yan G.G., Shen Z.W., Zhao W.S., Wang L., Zhang Y., Liu X.F., Sun G.S. (2023). Growth of 4H-SiC epitaxial layers at temperatures below 1500 °C using trichlorosilane (TCS). J. Cryst. Growth.

[19] Yang L., Zhao L.X., Wu H.W. (2020). Effect of temperature on conversion of basal plane dislocations to treading edge dislocations during 4H-SiC homoepitaxiy. J. Cryst. Growth.

[20] Sun Y.Q., Kang W.Y., Chen H.A., Chen X.L., Dong Y., Lin W., Kang J.Y. (2022). Selection of growth monomers on the 4H-SiC (0001) atomic step surfaces: From the first-principles calculations to homo-epitaxy verification. Appl. Surf. Sci..

[21] El Hageali S.A., Guthrey H., Johnston S., Norman A., Soto J., Odekirk B., Stahlbush R.E., Mahadik N.A., Gorman B.P., Al-Jassim M. (2023). Optoelectronic and structural characterization of trapezoidal defects in 4H-SiC epilayers and the effect on MOSFET reliability. J. Appl. Phys..

[22] Daigo Y., Watanabe T., Ishiguro A., Ishii S., Moriyama Y. (2021). Influence and Suppression of Harmful Effects Due to By-Product in CVD Reactor for 4H-SiC Epitaxy. IEEE Trans. Semicond. Manuf..

[23] Sun S., Song H., Yang J., Qu H., Wang W., Jian J. (2023). The etching behaviour of dislocations in N-doped 4H-SiC substrate. J. Cryst. Growth.

[24] Eto K., Mitani T., Momose K., Kato T. (2024). Propagation behaviour of threading screw dislocations during 4h-sic crystal growth using a hybrid method combined with solution growth and physical vapour transport growth on high-off-angle seeds. J. Cryst. Growth.

[25] Wang R., Huang Y., Yang D., Pi X. (2023). Impurities and defects in 4H silicon carbide. Appl. Phys. Lett..

[26] Hu J., Jia R., Xin B., Peng B., Wang Y., Zhang Y. (2016). Effect of low pressure on surface roughness and morphological defects of 4h-sic epitaxial layers. Materials.

[27] Hu J., Jia R., Niu Y., Zang Y., Pu H. (2019). Study of a new type nominal “washboard-like” triangular defects in 4H-SiC 4° off-axis (0001) Si-face homoepitaxial layers. J. Cryst. Growth.

[28] Liu X.S., Zhang J.R., Xu B.J., Lu Y.H., Zhang Y.Q., Wang R., Yang D.R., Pi X.D. (2022). Deformation of 4H-SiC: The role of dopants. Appl. Phys. Lett..

[29] Li J., Yang G., Liu X., Luo H., Xu L., Zhang Y., Cui C., Pi X., Yang D., Wang R. (2022). Dislocations in 4H silicon carbide. J. Phys. D Appl. Phys..

[30] Nishio J., Ota C., Iijima R. (2023). Partial dislocation structures at expansion terminating areas of bar-shaped single Shockley-type stacking faults and basal plane dislocations at the origin in 4H-SiC. Jpn. J. Appl. Phys..

[31] Kim H.-K., Kim S.I., Kim S., Lee N.-S., Shin H.-K., Lee C.W. (2020). Relation between work function and structural properties of triangular defects in 4H-SiC epitaxial layer: Kelvin probe force microscopic and spectroscopic analyses. Nanoscale.

[32] He Y., Yan G., Shen Z., Zhao W., Wang L., Liu X., Sun G., Zhang F., Zeng Y. (2020). Investigation of the distribution of deep levels in 4H-SiC epitaxial wafer by DLTS with the method of decussate sampling. J. Cryst. Growth.

[33] Gelczuk L., Dabrowska-Szata M., Kolkovsky V.I., Sochacki M., Szmidt J., Gotszalk T. (2020). Origin and anomalous behavior of dominant defects in 4H-SiC studied by conventional and Laplace deep level transient spectroscopy. J. Appl. Phys..

[34] Daigo Y., Ishiguro A., Moriyama Y., Mizushima I. (2021). Structure and reduction of large bumps formed on 4H-SiC epitaxial film originated from dislocations in substrate. J. Cryst. Growth.

[35] Chokawa K., Daigo Y., Mizushima I., Yoda T., Shiraishi K. (2021). First-principles and thermodynamic analysis for gas phase reactions and structures of the SiC (0001) surface under conventional CVD and Halide CVD environments. Jpn. J. Appl. Phys..

[36] Kodolitsch E., Kabakow A., Sodan V., Krieger M., Weber H., Tsavdaris N. (2023). Structural investigation of triangular defects in 4H-SiC epitaxial layers as nucleation source for bar shaped stacking faults (BSSFs). J. Phys. D Appl. Phys..

[37] Karhu R., Ghezellou M., Ul Hassan J. (2022). The Origin and Formation Mechanism of an Inclined Line-like Defect in 4H-SiC Epilayers. Phys. Status Solidi B Basic Solid State Phys..

[38] Ishiji K., Kato M., Sugie R. (2022). Characterization of Defect Structure in Epilayer Grown on On-Axis SiC by Synchrotron X-ray Topography. J. Electron. Mater..

[39] El Hageali S.A., Guthrey H., Johnston S., Soto J., Odekirk B., Gorman B.P., Al-Jassim M. (2022). Nondestructive microstructural investigation of defects in 4H-SiC epilayers using a multiscale luminescence analysis approach. J. Appl. Phys..

[40] Baierhofer D., Thomas B., Staiger F., Marchetti B., Förster C., Erlbacher T. (2022). Defect reduction in SiC epilayers by different substrate cleaning methods. Mater. Sci. Semicond. Process..

[41] Li R., Zhang K.M., Zhang Y., Zhang Z.Z., Ji P.X., Shi C.Q., Hao D.N., Zhang Y.P., Moro R., Ma Y.Q. (2022). Hydrogen etching of 4H-SiC(0001) facet and step formation. Mater. Sci. Semicond. Process..

[42] Remes Z., Stuchlik J., Stuchlikova T.H., Kupcik J., Mortet V., Taylor A., Ashcheulov P., Volodin V.A. (2020). Electroluminescence of thin film p-i-n diodes based on a-SiC: H with integrated Ge nanoparticles. Eur. Phys. J. Appl. Phys..

[43] Abadier M., Song H.Z., Sudarshan T.S., Picard Y.N., Skowronski M. (2015). Glide of threading edge dislocations after basal plane dislocation conversion during 4H-SiC epitaxial growth. J. Cryst. Growth.

